# Targeting the *ZMYM2-ANXA9* Axis with *FLT3* Inhibitor G749 Overcomes Oxaliplatin Resistance in Colorectal Cancer

**DOI:** 10.3390/biomedicines13051247

**Published:** 2025-05-20

**Authors:** Dezheng Lin, Yucheng Xu, Huanmiao Zhan, Yufan Liang, Riyun Liu, Jun Liu, Dandong Luo, Xiaochuan Chen, Jiawei Cai, Yifeng Zou

**Affiliations:** 1 Department of General Surgery, The Sixth Affiliated Hospital, Sun Yat-sen University, Guangzhou 510655, China; lindzh8@mail.sysu.edu.cn (D.L.);; 2 Guangdong Provincial Key Laboratory of Colorectal and Pelvic Floor Diseases, The Sixth Affiliated Hospital, Sun Yat-sen University, Guangzhou 515000, China; 3 Department of Pancreatic and Metabolic Surgery, Nanjing Drum Tower Hospital, Affiliated Hospital of Medical School, Nanjing University, Nanjing 210008, China; 4 Department of Urology, Kidney and Urology Center, Pelvic Floor Disorders Center, The Seventh Affiliated Hospital, Sun Yat-sen University, Shenzhen 518000, China; 5 Department of Clinical Medicine, The Sixth Clinical School of Guangzhou Medical University, Guangzhou 511436, China; 6 Department of Obstetrics and Gynecology, The Sixth Affiliated Hospital, Sun Yat-sen University, Guangzhou 515000, China

**Keywords:** *ANXA9*, *ZMYM2*, G749, colorectal cancer, chemoresistance

## Abstract

**Background:** Chemoresistance and tumor recurrence remain major obstacles in colorectal cancer (CRC) therapy. Elucidating the molecular mechanisms underlying treatment resistance is critical for improving therapeutic outcomes. **Methods**: We analyzed transcriptomic profiles from public datasets (TCGA and GSE39582) to identify differentially expressed genes associated with a poor response to neoadjuvant chemotherapy in CRC patients. Among 298 candidate genes, *ANXA9* emerged as significantly overexpressed in chemoresistant tumors and associated with a poor prognosis. These findings were further validated in an independent cohort of 146 Stage III CRC patients using immunohistochemistry and survival analysis. The expression of *ANXA9* was evaluated in oxaliplatin acquired-resistant CRC cell lines via qPCR and Western blot. Functional studies, including RNA interference, colony formation, apoptosis assays, and drug sensitivity testing, were performed in vitro and in vivo to assess the role of *ANXA9*. A high-throughput drug screen identified G749, a *FLT3* inhibitor, as a potential therapeutic agent. **Results:** *ANXA9* expression was significantly elevated in non-responders to chemotherapy and oxaliplatin-resistant CRC cell lines. The knockdown of *ANXA9* reduced proliferation and enhanced oxaliplatin sensitivity. G749 was found to suppress *ANXA9* expression in a dose-dependent manner and inhibit CRC cell growth in vitro and in patient-derived organoids. In a CRC xenograft mouse model, G749 reduced the tumor burden without observable toxicity. Mechanistically, we identified *ZMYM2* as a transcriptional regulator of *ANXA9*. ChIP-qPCR confirmed *ZMYM2* binding to the *ANXA9* promoter, especially in resistant cells. Silencing *ZMYM2* suppressed tumor cell growth and restored chemosensitivity. **Conclusions:** The *ZMYM2-ANXA9* signaling axis drives chemoresistance and tumor progression in CRC. *FLT3* inhibition by G749 effectively downregulates *ANXA9* and sensitizes tumors to chemotherapy, highlighting a novel therapeutic approach for chemoresistant CRC.

## 1. Introduction

Colorectal cancer (CRC) stands as a globally prevalent malignancy, distinguished not only by its incidence but also by a high propensity for recurrence [1]. However, the clinical landscape is increasingly complicated by recurrent disease and a rising tide of chemoresistance. These phenomena critically undermine the effectiveness of conventional treatments [2,3]. Therefore, it is an urgent need to delve into the molecular foundations of CRC chemoresistance. Previous studies have shown multiple mechanisms of CRC progression and therapy resistance [4,5,6,7,8]. Several clinical features contribute to chemotherapy resistance in CRC, including poor histologic differentiation, mismatch repair deficiency, and a high tumor mutational burden. These factors highlight that genetic and molecular interactions are critical barriers to effective tumor treatment [9,10,11,12]. Understanding resistance mechanisms in CRC is crucial for developing personalized therapies.

The availability of extensive genomic profiles in publicly accessible datasets has revolutionized cancer research. High-throughput data analysis now provides an efficient and cost-effective method, leveraging vast genomic information to accelerate discoveries and innovations in the field [13]. In this study, we identified *ANXA9* (Annexin A9) as highly correlated with poor treatment outcomes, including chemoresistance in CRC, suggesting its potential as a prognostic biomarker. Notably, *FLT3* (FMS-like tyrosine kinase 3) pathway inhibitors, such as G749, could downregulate *ANXA9* expression by inhibiting *ZMYM2*, potentially overcoming treatment failure in CRC patients.

Annexins, a family of Ca^2+^-dependent phospholipid-binding proteins, are classified into five subgroups (A–E). The Annexin A (ANXA) group, consisting of 13 members (*ANXA1-ANXA13*) found in various human organs, is increasingly implicated in tumor development and progression [14,15]. *ANXA9* is linked to metastasis in CRC, suggesting a poor prognosis [16,17,18,19,20,21]. In gastric cancer, its overexpression, correlating with immune infiltration, positions *ANXA9* as a potential novel biomarker [22,23]. However, the relationship between *ANXA9* expression and chemoresistance in CRC remains unexplored.

*FLT3*, a receptor tyrosine kinase essential for normal hematopoiesis and leukemic cell proliferation, is highly expressed in leukemic cells. Targeting *FLT3* is crucial in acute myeloid leukemia (AML) treatment, with inhibitors like midostaurin, gilteritinib, and quizartinib available. These inhibitors block *FLT3* kinase activity, suppress leukemic growth, and improve survival rates. The FDA approved midostaurin for newly diagnosed and gilteritinib for relapsed or refractory *FLT3*-mutated AML in 2017 [24,25]. While *FLT3* inhibitors have been extensively investigated in hematological malignancies, emerging studies have begun to explore their broader biological functions, including the regulation of oncogenic signaling in solid tumors. *FLT3* signaling modulates PI3K/AKT and MAPK pathways, which are frequently dysregulated in CRC—and may influence tumor behavior via epigenetic or transcriptional mechanisms [26,27,28,29].

Importantly, our data revealed that *ZMYM2*, a transcriptional regulator in the *FLT3* pathway, is strongly associated with *ANXA9* expression in CRC. *ZMYM2* is a transcriptional regulator involved in chromatin remodeling and gene expression regulation. It is a component of several transcriptional repression complexes, such as the histone deacetylase complex, which influences tumor biology by modulating the accessibility of chromatin [30]. These findings provide a rationale for repurposing *FLT3* inhibitors in CRC by targeting a previously underappreciated epigenetic axis.

In this study, we identified *FLT3* inhibitor G749 as a sensitizer to oxaliplatin through drug screening, demonstrating robust antitumor effects both in vitro and in vivo models. Our results reveal a mechanism underlying oxaliplatin resistance and suggest that targeting the *ZMYM2*/*ANXA9* axis with G749 may offer a therapeutic strategy to overcome chemotherapy resistance in CRC patients.

## 2. Materials and Method

### 2.1. Human Specimens

Tumor tissues were collected from CRC patients undergoing surgery at the Sixth Affiliated Hospital, Sun Yat-sen University. Informed consent was obtained from all patients, and the study was approved by the Medical Ethics Committee of the Sixth Affiliated Hospital, Sun Yat-sen University. A pathologist assessed the tumor tissues to confirm the type and grade of the tumors. Tumors’ pT, pN, and pM status were assessed according to the Seventh Edition of the AJCC staging criteria [31]. The Institutional Review Board of the Sixth Affiliated Hospital, Sun Yat-sen University, approved the collection of data (approval number: 2022ZSLYEC-161).

### 2.2. Mice

Six- to eight-week-old female BALB/c-nude were procured from Charles River Laboratories (Beijing, China) and maintained at the Biological Resource Center. A minimum of five mice per group were used in all experiments. Tumor size was measured every four days and calculated as V = (d^2^ × D)/2, where d is the minor axis and D is the major axis. Tumor volume is reported as mass (mm^3^, mean ± SD.). Measurements were taken prior to cage identification. Sample sizes were not predetermined statistically. When the tumors reached a volume of 70 mm^3^, the mice were randomized into two groups with similar tumor sizes: a control group treated with normal saline (n = 6, gastric gavage) and a group receiving G749 (n = 6, 80 mg kg^−1^, every day, gastric gavage). The mice were housed in specific pathogen-free conditions and handled according to the guidelines of the Animal Care and Use Committee of Sun Yat-sen University.

### 2.3. Cell Culture

All cancer cell lines DLD1, HCT116, and WiDr were acquired from the American Type Culture Collection (Manassas, VA, USA) and authenticated using short tandem repeat analysis. All cell lines were verified to be mycoplasma-free using a commercial detection kit (2523348, Bulldog Bio, Portsmouth, NH, USA). Cells were maintained in DMEM or RPMI medium (Thermo Fisher Scientific, Waltham, MA, USA) supplemented with 10% fetal bovine serum or 10% dialyzed human serum and maintained at 37 °C in a humidified atmosphere of 5% CO_2_ and 95% air.

### 2.4. Clonogenic Survival Assays

Cells (100–500) were seeded in 6- or 12-well plates and allowed to adhere for 24 h before treatment with DMSO or the indicated drugs. Following drug treatment, the cells were cultured for 7–14 days to allow colony formation. Colonies were rinsed twice with PBS to remove non-adherent cells, fixed with 4% formaldehyde in PBS for 15 min, and stained with 0.1% crystal violet in 10% ethanol for 20 min. After staining, the solution was aspirated, and the colonies were washed three times with water, air-dried, and imaged using a scanner (Epson Perfection V700 Photo, Suwa, Japan). A minimum of three biological replicates were performed.

### 2.5. Drugs

Oxaliplatin was sourced from Selleck (Houston, TX, USA), while G749 was obtained from Topscience (Shanghai, China).

### 2.6. ChIP–Quantitative PCR

To assess the binding activity of *ZMYM2* to the *ANXA9* promoter region, a ChIP assay was performed using the SimpleChIP Plus Enzymatic Chromatin IP Kit (#9005, CST), according to the manufacturer’s instructions, for 8024 cells. Briefly, 1 × 10^7^ cells were cultured for 24 h, washed twice with PBS, crosslinked with formaldehyde, lysed in sodium dodecyl sulfate buffer, and sonicated. The fragmented chromatin was then diluted in ChIP buffer and incubated overnight with anti-HIF1α (CST, 36169, Danvers, MA, USA), anti-HIF2α monoclonal antibody (#59973, CST, Danvers, MA, USA), or anti-ZMYM2 (MABN2263, Millipore, Burlington, MA, USA). A normal rabbit IgG (#2729, CST, Danvers, MA, USA) served as a negative control, while histone H3 rabbit monoclonal antibody was used as a positive control. Immunoprecipitated chromatin was collected after incubation with protein A/G magnetic beads. The bound chromatin was eluted, digested with proteinase K, and purified for RT-qPCR analysis [32].

### 2.7. Flow Cytometric Analysis

A total of 2 × 10^5^ cells were treated with the indicated drugs for 72 h. After treatment, the cells were washed with ice-cold PBS and stained using the Annexin V–FITC Apoptosis Detection Kit (Vazyme, Nanjing, China), following the manufacturer’s instructions. The stained cells were then analyzed by flow cytometry (SP6800, Sony, Tokyo, Japan) [32].

### 2.8. Western Blotting 

Cultured cells were harvested using a scraper and lysed on ice in RIPA buffer (P0013B, Beyotime, Shanghai, China) containing 50 mM Tris-HCl (pH 7.4), 150 mM NaCl, 1% Triton X-100, 1% sodium deoxycholate, and 0.1% SDS, supplemented with phosphatase and protease inhibitors. After 15 min of incubation on ice, the lysates were sonicated and centrifuged at 12,000× *g* for 20 min. Protein extraction from nuclear and cytoplasmic compartments (P0028, Beyotime, Shanghai, China), as well as membrane and cytosolic fractions (P0033, Beyotime, Shanghai, China), was carried out according to the manufacturers’ instructions. The resulting cell lysates were quantified using a BCA Protein Assay Kit (20201ES90, Yeasen, Shanghai, China). Equal amounts of protein were denatured at 100 °C for 10 min, resolved on 8–12% SDS-PAGE alongside a prestained protein marker (M221, GenStar, Beijing, China), and transferred to 0.45 μm PVDF membranes (IPVH00010, Millipore, Burlington, MA, USA). The membranes were blocked for 2 h at room temperature in a buffer containing 5% skim milk (wt/vol) in TBST (10 mM Tris-HCl [pH 7.5], 500 mM NaCl, 0.1% Tween 20). After blocking, the membranes were incubated overnight at 4 °C with primary antibodies. The next day, the membranes were washed three times in TBST for 10 min each, incubated for 1 h at room temperature with HRP-conjugated secondary antibodies diluted in blocking buffer, and washed again in TBST three times. Protein bands were visualized using enhanced chemiluminescence (P10300, NCM Biotech, Suzhou, China) and detected with the Tanon 2500 Luminescence Imaging System (Tanon, Shanghai, China). The antibodies used in this study are anti-ANXA9 (15416-1-AP, 1:5000, Proteintech, San Diego, CA, USA), anti-ZMYM2 (MABN2263, Millipore, Burlington, MA, USA) and anti-α-tublin (ab6160, Abcam, Cambridge, UK) [32].

### 2.9. Immunohistochemistry Staining

After deparaffinization, antigen retrieval was performed using basic EDTA antigen retrieval buffer (pH 9.0, Panovue, Shanghai, China) in a microwave, followed by two washes with TBS. Sections were pre-incubated with hydrogen peroxide, blocked with goat serum (Beyotime, Shanghai, China), and incubated with primary antibodies against *ANXA9* (15416-1-AP, 1:200, Proteintech, San Diego, CA, USA) and Ki67 (ZM-0166, ZSGB-Bio, Beijing, China) overnight at 4 °C. The next day, the sections were rewarmed and washed three times with TBS. According to the Dako REAL™ EnVision™ Detection System kit protocol (Dako, Glostrup, Denmark), the sections were incubated with rabbit/mouse secondary antibodies for 30 min, followed by DAB staining and hematoxylin counterstaining. All sections were evaluated by two experienced pathologists using the Immune Response Score (IRS) system [33]. Staining intensity was scored as follows: 1 (no staining), 2 (weak), 3 (moderate), and 4 (strong). The percentage of positive tumor cells was scored as 0 (<6%), 1 (6–25%), 2 (26–50%), 3 (51–75%), and 4 (>75%). Histochemistry (H)-scores (0–16) were calculated by multiplying the staining intensity score by the percentage of positive cells [34].

### 2.10. RNA Interference

Negative control siRNA and siRNAs targeting human *ANXA9* and *ZMYM2* were synthesized by Sangon Biotech (Shanghai, China). Transfections were performed using Lipofectamine RNAiMAX reagent (Thermo Fisher Scientific) according to the manufacturer’s instructions [32]. The sequence of used *ANXA9* siRNAs can be found in Table S1.

### 2.11. Real-Time Quantitative PCR

Total RNA was extracted using TRIzol reagent and further purified with the RNAeasy Mini Kit (Qiagen, Hilden, Germany). Reverse transcription and quantitative PCR were conducted using the TransScript All-in-One First-Strand cDNA Synthesis SuperMix for qPCR (Transgene Biotech, Beijing, China) and KAPA SYBR FAST qPCR Master Mix (2×) (Sigma-Aldrich, St. Louis, MO, USA). mRNA levels were normalized to 18S rRNA or GAPDH as an internal control. Reactions were performed and analyzed using a Real-Time PCR Detection System (Bio-Rad, Hercules, CA, USA). All experiments were conducted in biological triplicates unless otherwise specified [32]. The specific primers used for *ANXA9* can be found in Table S1.

### 2.12. Organoid Culture

Murine intestinal organoids were generated as previously described. Intestinal tissue was harvested from CRC patients and washed with cold PBS to remove debris. Crypts were isolated by incubation in 10 mM EDTA at 4 °C for 30 min, followed by mechanical dissociation and filtration through a 70 μm strainer. Isolated crypts were counted and resuspended in Matrigel (Corning Inc., Corning, NY, USA) at a density of 500 crypts per 50 μL dome. The Matrigel domes were plated in pre-warmed 24-well plates and polymerized at 37 °C for 10 min. Following polymerization, 500 μL of complete organoid medium (Advanced DMEM/F12 supplemented with 50 ng/mL EGF, 100 ng/mL Noggin, and 500 ng/mL R-spondin) was added to each well. Organoids were maintained in a humidified 37 °C incubator with 5% CO_2_, and the media were replaced every two days. Organoids typically formed within 3–5 days. Once established, the organoids were divided into two groups: the control group was treated with DMSO, and the experimental group received G749 (μM). Both treatments were administered every 3 days for 10 days. Passaging was performed weekly by mechanical disruption and replating in fresh Matrigel. All organoid cultures were handled under sterile conditions [32].

### 2.13. Statistical Analysis

Data are presented as the mean ± SD from three independent experiments. Statistical differences between two groups were evaluated using a two-tailed Student’s *t*-test, while comparisons among more than two groups were analyzed using a two-way ANOVA with Bonferroni’s correction in GraphPad Prism 7. A *p*-value of <0.05 was considered statistically significant.

### 2.14. Replicates and Experimental Design

Unless otherwise specified, all in vitro experiments were independently repeated three times (biological replicates), with each experiment performed in technical triplicate. For colony formation, qPCR, flow cytometry, and Western blot assays, representative data from three biological replicates are presented.

In vivo xenograft studies were conducted with 5–8 mice per group, based on preliminary effect size observations and in accordance with previously published protocols. This sample size was chosen to ensure sufficient statistical power (β > 0.8, α = 0.05) while adhering to ethical standards under institutional animal care guidelines.

Immunohistochemical staining was assessed on tumor sections from biological replicates, with two technical replicates per marker evaluated independently by two pathologists. Organoid culture experiments were performed in three biological replicates unless otherwise noted. All animal experiments were approved by the Institutional Animal Care and Use Committee (IACUC), and every effort was made to minimize animal usage while ensuring data reproducibility.

## 3. Result

### 3.1. Elevated ANXA9 Expression Is Correlated with Chemoresistance and Poor Prognosis

**Figure 1:**
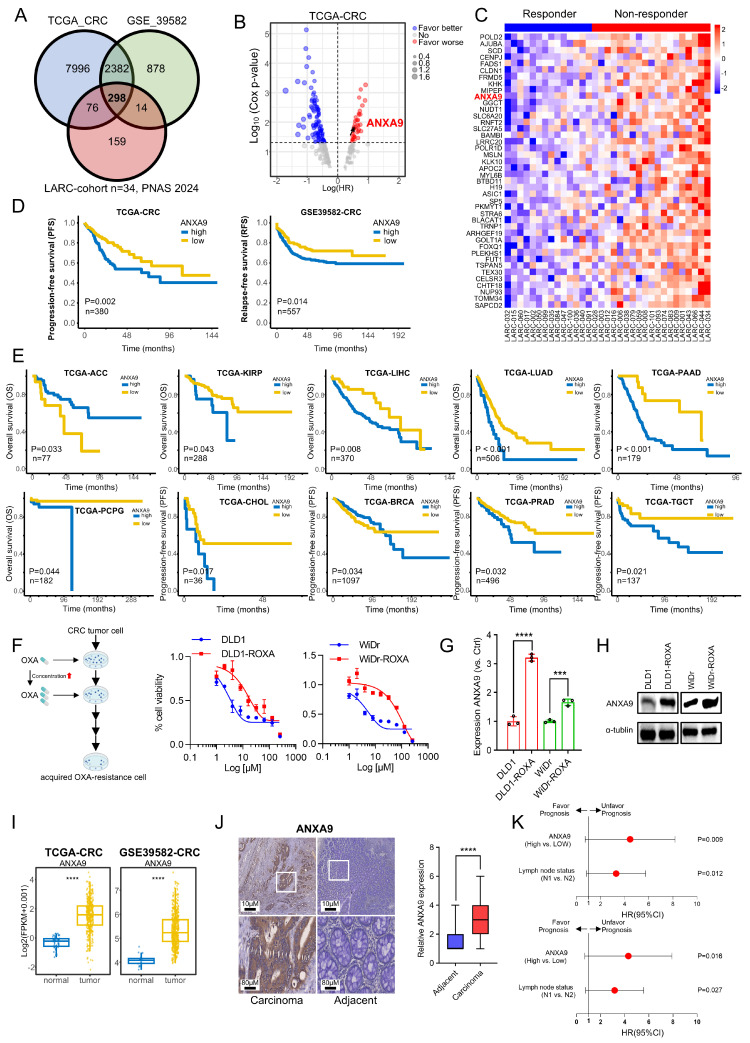
Elevated *ANXA9* expression is correlated with chemoresistance and a poor prognosis. (**A**) The intersection of genes with high expression levels in CRC, identified from both the TCGA and GSE39582 databases, as well as genes with elevated expression in non-responders to LARC neo-adjuvant therapy (PNAS 2024). (**B**) Prognostic analysis of the intersecting genes in the TCGA CRC dataset reveals a notable correlation between elevated *ANXA9* expression and a poor prognosis. (**C**) Heatmap illustrating differentially expressed genes (DEGs) with elevated expression levels associated with a poor prognosis between responder and non-responder groups. (**D**) Stratified prognostic analysis based on high and low *ANXA9* expression levels in the TCGA and GSE39582 datasets. (**E**) Overall survival (OS) curves stratified by high and low *ANXA9* expression across various cancer types in the TCGA dataset. (**F**) DLD1, DLD1-ROXA, WiDr, and WiDr-ROXA cells were subjected to the indicated concentrations of oxaliplatin, and viable cell numbers were assessed after 96 h. Data represent the mean ± SD of three independent biological replicates, each with three technical replicates. (**G**) qPCR analysis demonstrates higher *ANXA9* mRNA expression in oxaliplatin-resistant CRC cells versus oxaliplatin-sensitive CRC cells. qPCR results represent the mean ± SD of three biological replicates, each with technical triplicate measurements. *t*-test was used for statistical comparisons. (**H**) Western blot analysis shows increased *ANXA9* protein levels in oxaliplatin-resistant CRC cells relative to oxaliplatin-sensitive CRC cells. (**I**) *ANXA9* expression is markedly elevated in CRC tumor tissues versus normal tissues. Representative Western blots from three independent biological replicates are shown. Densitometry quantifications (if included) are based on normalized values across replicates. (**J**) Immunohistochemistry (IHC) images depicting *ANXA9* expression in CRC patient samples (n = 146). (**K**) Correlation between pathological characteristics and overall survival (OS) in the CRC cohort, as assessed by multivariate (**upper**) and univariate (**lower**) Cox regression analyses. In the figure caption, “***” means *p* < 0.001, and “****” means *p* < 0.0001.

We identified 298 candidate genes associated with chemoresistance for further investigation (Figure 1A). Deeper investigation revealed that only a subset of these genes correlated with a poor prognosis in CRC, as per TCGA data, with *ANXA9* showing a strong association with adverse outcomes (Figure 1B). The expression levels of the identified genes were shown in both responders and non-responders (Figure 1C).

An elevated *ANXA9* expression correlated with a poor prognosis in CRC patients from the TCGA and GSE39582 cohorts (Figure 1C). Then, we expanded our research to explore the link between *ANXA9* expression and cancer prognosis. Analyzing TCGA data, we observed that elevated *ANXA9* levels were associated with poor outcomes in several cancers (Figure 1E). These findings indicate that *ANXA9* might serve a critical function in the progression of multiple cancer types, highlighting its potential as a universal biomarker and therapeutic target in oncology. To further elucidate the role of *ANXA9* in the chemoresistance of CRC patients, we analyzed its expression in both CRC cell lines and oxaliplatin acquired-resistant CRC cell lines. We established an oxaliplatin acquired-resistant cell line model (DLD1-ROXA and WiDr-ROXA), confirmed by a growth inhibition assay (IC50; Figure 1F). Using quantitative PCR (qPCR) and WB analyses, we confirmed an elevated expression of *ANXA9* in oxaliplatin acquired-resistant CRC cell lines (Figure 1G,H). Our study demonstrates that *ANXA9* levels are significantly elevated in CRC tumor tissues compared to normal tissues (Figure 1I).

To investigate the clinical significance of *ANXA9* in CRC, we assembled an in-house cohort of 146 Stage III CRC patients. Consistent with the above experimental results, *ANXA9* expression was markedly elevated in CRC tumors compared to normal tissues (Figure 1J). Based on the patients mentioned above, we further explored the association between *ANXA9* expression and the clinical characteristics of CRC patients. We observed that a higher positivity rate of *ANXA9* expression was associated with a greater tumor infiltration depth and a higher incidence of lymphatic metastasis in CRC tissue samples. (*p* < 0.05, Table S2). Moreover, univariate and multivariate Cox regression analyses identified *ANXA9* expression as an independent prognostic factor in these patients (Figure 1K).

### 3.2. High Expression of ANXA9 Promotes Malignant Phenotypes in CRC Cells

**Figure 2:**
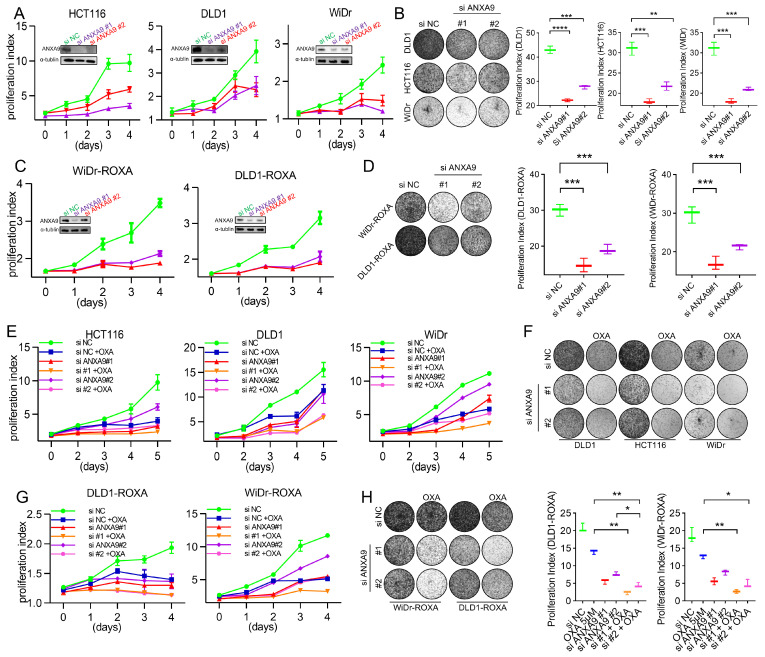
*ANXA9* promotes malignant phenotypes in CRC cells. (**A**) Viability of CRC cell lines following *ANXA9* silencing or control treatment. Data represent the mean ± SD of three independent biological replicates, each performed in technical triplicate. Statistical significance was assessed using an unpaired two-tailed Student’s *t*-test. (**B**) Colony formation assay of CRC cells with *siANXA9* silencing after 12 days (**left**) and relative colony formation (**right**). Quantification is based on three biological replicates. Data are shown as the mean ± SD. One-way ANOVA with Tukey’s post hoc test was used to evaluate statistical significance. (**C**) Viability of CRC-ROXA cell lines following *ANXA9* silencing or control treatment. Results represent the mean ± SD from three independent biological replicates, with technical triplicates. Statistical comparisons were made using *t*-test. (**D**) Colony formation assay of CRC-ROXA cells with *ANXA9* silencing after 12 days (**left**) and relative colony formation (**right**). Data shown as the mean ± SD of three biological replicates. Statistical analysis performed using one-way ANOVA. (**E**) Growth curves of CRC cells with *ANXA9* depletion and OXA treatment over 5 days. Error bars represent mean ± SD from three biological replicates. OXA was used at the specified concentrations; a *t*-test was applied for pairwise comparisons at each timepoint. (**F**) Colony formation assays were conducted to evaluate CRC cells treated with *siANXA9*, OXA, or their combination. OXA was applied at concentrations of 2.5 μM for DLD1, 1.5 μM for HCT116, and 2 μM for WiDr cells. Colony formation was assessed after 12 days of treatment. In the growth curve assay for the DLD1, HCT116, and WiDr cells, OXA was used at the same concentrations. Data are presented as the mean ± SD from three biological replicates. Significance was determined using one-way ANOVA. (**G**) Growth curves of CRC-ROXA cells with *ANXA9* depletion and OXA treatment over 5 days. Error bars indicate the mean ± SD of three independent biological replicates. (**H**) Colony formation assay of CRC-ROXA cells following *ANXA9* silencing, oxaliplatin treatment, or their combination at 12 days (**left**) and relative colony formation (**right**). OXA was applied at concentrations of 5 μM for DLD1-ROXA and WiDr-ROXA cells. In the growth curve assay for DLD1-ROXA and WiDr-ROXA cells, OXA was used at the same concentrations. Colony assay results are shown as the mean ± SD of three biological replicates. Statistical comparisons were conducted using one-way ANOVA. In the figure caption, “*” means *p* < 0.05, “**” means *p* < 0.01, “***” means *p* < 0.001, and “****” means *p* < 0.0001.

We next investigated the functional relevance of *ANXA9* in CRC. The biological role of *ANXA9* was investigated in selected CRC cell lines through genetic knockdown by small interfering RNA (siRNA), with the depletion effect confirmed by WB. CRC cells transfected with *ANXA9* siRNA exhibited a significantly reduced colony formation and cell proliferation (Figure 2A,B). More importantly, the same findings can be observed in oxaliplatin-resistant CRC cells (Figure 2C,D), suggesting that targeting *ANXA9* might reverse drug resistance in CRC.

Given the critical impact of chemotherapy failure on tumor recurrence in Stage II–III CRC patients, enhancing chemosensitivity presents itself as a promising therapeutic strategy. To explore the pharmacological value of targeting *ANXA9*, CRC cells were treated with oxaliplatin following siRNA transfection. The proliferation rate of cells transfected with *ANXA9* siRNA and treated with oxaliplatin was significantly lower compared to cells receiving only the single treatment (Figure 2E,F). More importantly, similar observations were made in oxaliplatin acquired-resistant CRC cells (Figure 2G,H). This indicates that targeting *ANXA9* may have the potential to overcome drug resistance in CRC. Functional studies revealed that *ANXA9* knockdown via siRNAs significantly inhibited proliferation and colony formation in both oxaliplatin-sensitive and -resistant CRC cells. These findings suggest that targeting *ANXA9* could enhance oxaliplatin sensitivity and offer a promising therapeutic approach against chemoresistant CRC.

### 3.3. FLT3 Inhibitor G749 Suppresses the Expression of ANXA9

**Figure 3:**
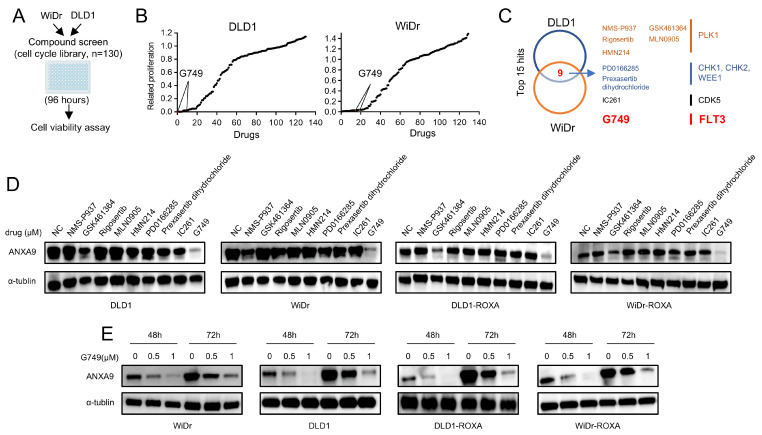
*ANXA9* promotes malignant phenotypes in CRC cells. (**A**) Schematic representation of the drug screening procedure: DLD1 and WiDr cells were seeded into 96-well plates and treated with 130 kinase inhibitors targeting cell cycle pathways (1.0 μM) during the primary screening phase. (**B**) Summary of drug screening results in WiDr and DLD1 cell lines treated with 130 compounds, ranked by efficacy, with *FLT3* pathway inhibitors (G749) highlighted in red. (**C**) Identification of nine compounds effective in both WiDr and DLD1 cell lines through screening. (**D**) WB analysis of *ANXA9* protein levels in CRC cells subjected to the nine identified compounds for 48 h. (**E**) WB analysis of *ANXA9* protein expression in CRC cells subjected to G749 for 48 or 72 h. Western blots represent results from three biological replicates.

We performed a high-throughput drug screening experiment on CRC cell lines, aiming to find drugs that can effectively kill tumor cells. According to our experimental results, we found that nine of the top fifteen target drugs overlapped in their efficacy profiles. This observation underscores the potential effectiveness of inhibitors targeting PLK1, CHK1, CHK2, CDK5, and *FLT3* in concurrently suppressing the proliferation of both DLD1 and WiDr cells (Figure 3A–C, details are provided in Tables S3–5). Among the nine drugs identified, we treated CRC cells with each for 48 h and conducted WB analyses to detect *ANXA9* expression. Our results indicate that only G749 effectively downregulated *ANXA9* expression in both oxaliplatin-sensitive and -resistant CRC cells (Figure 3D). To further explore the potential of G749 in modulating *ANXA9* expression, we performed WB analyses on CRC cells treated with varying concentrations of G749. Our findings confirm that G749 suppresses *ANXA9* expression in a dose-dependent manner. Notably, this suppressive effect is also observed in chemoresistant CRC cells, suggesting a consistent mechanism of action across different cellular contexts (Figure 3E). These findings suggest a promising therapeutic potential for G749 in overcoming oxaliplatin resistance through the modulation of the *ANXA9* pathway.

### 3.4. G749 Inhibits Proliferation of CRC Tumor Both In Vitro and In Vivo

**Figure 4:**
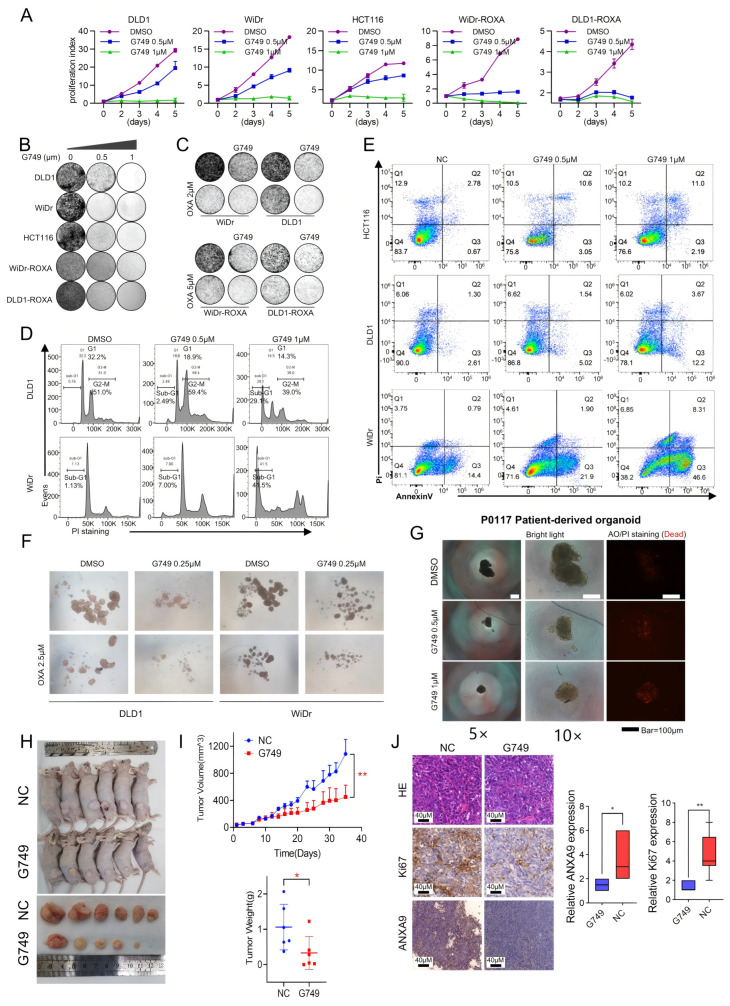
G749 inhibits the proliferation of CRC tumor both in vitro and in vivo. (**A**) Growth curves of DLD1, HCT116, WiDr, DLD1-ROXA, and WiDr-ROXA cells subjected to G749 are presented. Data are expressed as the mean ± SD (n = 3). Data are expressed as the mean ± standard deviation (SD) from three independent biological replicates, each performed in technical triplicate. An unpaired two-tailed Student’s *t*-test was used for statistical comparisons at each timepoint. (**B**) Representative images of colony formation assays in oxaliplatin-resistant and oxaliplatin-sensitive CRC cells treated with either DMSO or *FLT3* inhibitors are shown. Quantification (if applicable) was based on three biological replicates. Data are shown as the mean ± SD. Statistical analysis was performed using one-way ANOVA. (**C**) Representative images of colony formation assays in oxaliplatin-resistant and oxaliplatin-sensitive CRC cells subjected to DMSO, OXA, G749, or their combination are displayed. G749 was applied at concentrations of 0.25 μM for WiDr, 0.5 μM for DLD1, 0.5 μM for WiDr-ROXA, and 0.25 μM for DLD1-ROXA cells. Colony assay data are from three biological replicates and expressed as the mean ± SD. Statistical significance was assessed using one-way ANOVA. (**D**) Flow cytometry analysis using PI staining indicates that G749 treatment significantly increased apoptosis rates in CRC cells, with apoptosis levels positively correlating with drug concentrations. Results represent three biological replicates. Error bars indicate the mean ± SD. Statistical analysis was performed using one-way ANOVA. (**E**) Flow cytometry analysis using PI and Annexin V staining demonstrates a concentration-dependent increase in apoptosis in CRC cells following G749 treatment. Data are shown as the mean ± SD of three biological replicates. Significance was assessed using one-way ANOVA with Tukey’s post hoc test. (**F**) Representative images of tumor sphere formation assays in DLD1 and WiDr cells subjected to DMSO, G749, OXA, or their combination are provided (scale bar, 500 μm). Quantification reflects three independent biological replicates. Data are presented as the mean ± SD. (**G**) Representative images of P0117 organoids subjected to DMSO or G749 are shown. Organoid viability and morphology were assessed in three independent biological replicates unless otherwise indicated. (**H**,**I**) The in vivo efficacy of oxaliplatin and G749 was evaluated by monitoring tumor volume growth curves, tumor weight, and mouse body weight in DLD xenograft models. Error bars indicate the mean ± standard error of the mean (SEM) from 6–8 mice per group. Statistical comparisons were performed using a repeated-measures ANOVA (for tumor volume curves) and Student’s *t*-test (for tumor weight). (**J**) Representative images of hematoxylin–eosin (HE) staining and immunohistochemistry (IHC) for Ki-67 and *ANXA9* in DLD1 xenograft tumors are shown on the left. Quantification of Ki-67 and *ANXA9* expression levels in two groups of DLD1 xenograft tumors is provided on the right. Data are shown as the mean ± SD of three biological replicates. Differences were analyzed using an unpaired Student’s *t*-test. In the figure caption, “*” means *p* < 0.05, “**” means *p* < 0.01.

To investigate the effect of G749 on CRC cells, we conducted a series of functional experiments. Our research explored the effects of G749 on the proliferation of CRC cells and oxaliplatin acquired-resistant CRC cells. Initial observations indicated that G749 exerts a dose-dependent inhibitory influence on the proliferation of CRC cells, with the effect becoming more pronounced at higher drug concentrations (Figure 4A,B). More importantly, G749 can enhance the sensitivity to oxaliplatin, whether in oxaliplatin-sensitive or -resistant CRC cells (Figure 4C). A significant relationship exists between apoptosis and tumor drug resistance. Consequently, we delved deeper into the relationship between G749 and cellular apoptosis. Notably, G749 treatment significantly increased apoptosis in CRC cells, as determined through advanced flow cytometry techniques. Notably, there is a positive association between the concentration of G749 and the number of apoptotic cells (Figure 4D,E). These findings significantly enhance our understanding of the therapeutic potential of G749 in combating CRC, suggesting its efficacy in promoting apoptotic pathways.

We next assessed the functional impact of G749 treatment on CSCs in CRC. CRC cells were cultured in serum-free medium to facilitate sphere formation, a characteristic property of CSCs. Following treatment with G749, there was a significant inhibition of tumor sphere formation. Importantly, the degree of suppression showed a positive association with the concentration of G749. Additionally, G749 treatment enhanced the sensitivity of these cells to chemotherapy drugs (Figure 4F), indicating its potential as a therapeutic agent targeting CSC-associated chemoresistance in CRC. More importantly, treatment with G749 significantly suppressed the growth of patient-derived organoids from individuals with recurrent CRC tumors (Figure 4G). These findings suggest that G749 may exert anti-proliferative effects in oxaliplatin-resistant CRC models, warranting further investigation in recurrent disease contexts.

To further validate the combinatory effects in vivo, we investigated the anti-tumor efficacy of G749 using a DLD1-derived xenograft mouse model. G749 treatment resulted in a significantly enhanced anti-tumor effect (Figure 4H,I), as demonstrated by a marked reduction in Ki-67 and *ANXA9* expression levels, assessed through immunohistochemistry (IHC) analysis (Figure 4J). Concurrently, no significant changes in body weight were observed in G749-treated mice, indicating good tolerability under the tested dosing regimen (Figure 4I). Collectively, these findings underscore the potential clinical relevance of G749 for treating CRC patients.

### 3.5. ZMYM2 Drives the Malignant Phenotypes in CRC Cells

**Figure 5:**
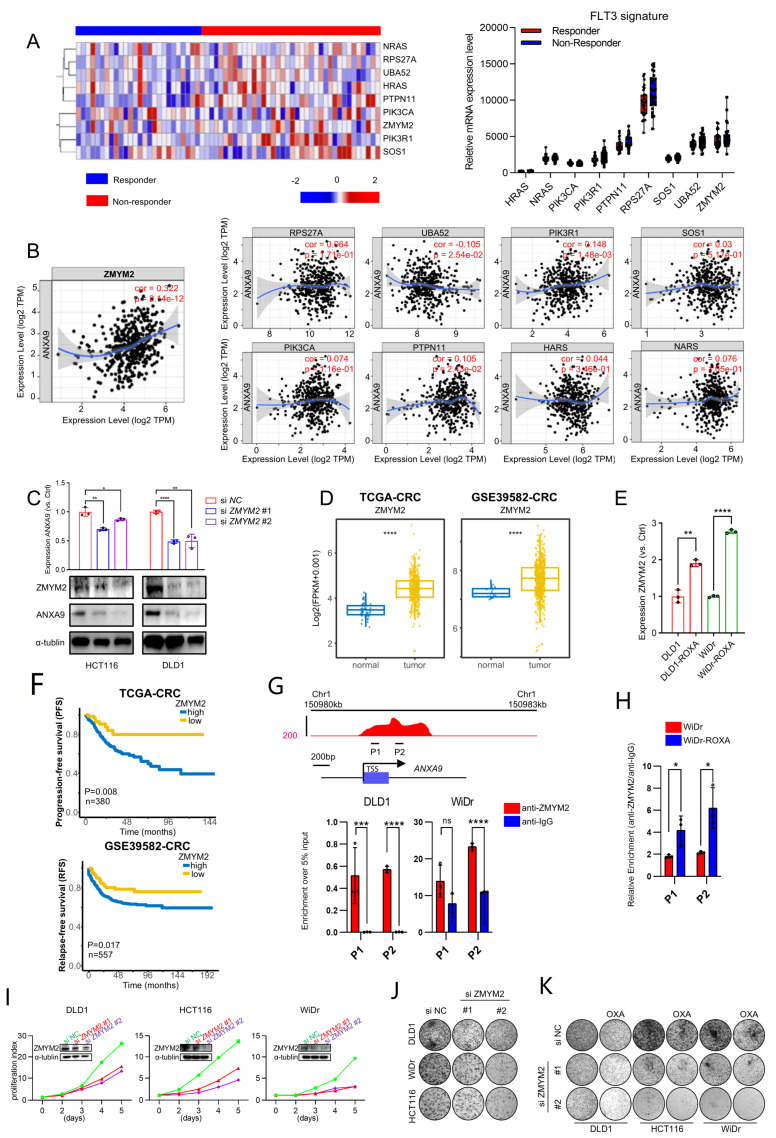
*ZMYM2* drives the malignant phenotypes in CRC cells. (**A**) Expression of the *FLT3* pathway signature in neoadjuvant-treated CRC patients: a comparison between responders and non-responders. (**B**) Correlation between the *FLT3* pathway signature and *ANXA9* expression in CRC. (**C**) Through qPCR and WB experiments, the knockdown of *ZMYM2* in CRC cells downregulated *ANXA9* expression. qPCR and Western blot data are from three independent biological replicates, each with technical triplicates. Statistical tests: *t*-test. (**D**) Analysis of TCGA and GSE3958 datasets revealed the expression levels of *ZMYM2* in CRC. (**E**) qPCR analysis demonstrates higher *ZMYM2* mRNA expression in oxaliplatin-resistant CRC cells compared to oxaliplatin-sensitive CRC cells. qPCR results are presented as mean ± SD from three biological replicates, each measured in technical triplicates. Statistical comparisons were performed using a *t*-test. (**F**) Overall survival (OS) curves for patients grouped by high and low *ZMYM2* expression levels in CRC from the TCGA and GSE39582 datasets. (**G**) A schematic diagram illustrates the locations of ChIP primers (P1–P2) across the *ANXA9* promoter region (**upper panel**). Chromatin extracts from DLD1, WiDr, and WiDr-ROXA cells were subjected to ChIP using either an anti-ZMYM2 antibody or normal IgG as a control. The enrichment of *ZMYM2* at the *ANXA9* promoter was assessed by qPCR (**lower panel**). Results are normalized to input controls and presented as the mean ± standard deviation (SD) of technical triplicates. (**H**) ChIP-qPCR analysis comparing *ZMYM2* binding to the *ANXA9* promoter between oxaliplatin-sensitive and -resistant CRC cell lines. Chromatin extracts were immunoprecipitated with anti-ZMYM2 antibody, and qPCR was used to quantify promoter binding. Data are shown as the mean ± SD of technical triplicates, normalized to input. Due to limited chromatin availability, ChIP was performed on one biological replicate per cell line, but the results were reproducible in independent parallel experiments. An unpaired two-tailed Student’s *t*-test was used for pairwise comparisons, and statistical significance reflects differences in *ZMYM2* binding between the groups. (**I**) Viability of CRC cell lines following *ZMYM2* silencing compared to control treatment. Data are shown as the mean ± SD of three independent biological replicates, each performed in technical triplicate. Statistical comparisons were made using an unpaired two-tailed Student’s *t*-test. (**J**) Colony formation assay of CRC cells following *ZMYM2* silencing, observed after 12 days. Results represent the mean ± SD from three biological replicates. Statistical significance was determined using a one-way ANOVA with Tukey’s post hoc test. (**K**) Colony formation assay of CRC cells subjected to si *ZMYM2*, OXA treatment, or their combination, assessed at 12 days. OXA was applied at concentrations of 4 μM for DLD1, 2.5 μM for HCT116, and 1.5 μM for WiDr cells. Colony formation data are presented as mean ± SD from three biological replicates. Significance was assessed using one-way ANOVA. In the figure caption, “*” means *p* < 0.05, “**” means *p* < 0.01, “***” means *p* < 0.001, and “****” means *p* < 0.0001.

To further investigate the *FLT3* pathway, we analyzed the expression profile of its signature in responder and non-responder groups’ samples (Figure 5A). Further analysis revealed a significant correlation between the *FLT3* pathway signature and *ANXA9* expression in CRC. Notably, *ZMYM2* exhibited the strongest correlation with *ANXA9*, suggesting a close regulatory relationship between these molecules (Figure 5B). Subsequently, we investigated the functional relationship between *ZMYM2* and *ANXA9* in CRC cell lines. Our experiments demonstrated that the depletion of *ZMYM2* resulted in a marked reduction in *ANXA9* expression (Figure 5C). These results provide direct evidence that *ZMYM2* transcriptionally regulates *ANXA9* expression, further supporting the notion that the ZMYM2-*ANXA9* axis is a critical pathway in the progression of CRC and a potential target for overcoming chemotherapy resistance. Given the role of *ZMYM2* as a transcriptional regulator, we suggest that its inhibition disrupts the downstream expression of *ANXA9*, thereby impairing the survival and proliferation of CRC cells. This mechanistic insight underscores the possibility of targeting the *ZMYM2-ANXA9* axis as a therapeutic strategy to overcome chemotherapy resistance in CRC.

We further explored the role of *ZMYM2* in the development and progression of CRC. Our findings reveal that *ZMYM2* expression is significantly elevated in colorectal tumor tissues compared to adjacent normal tissues (Figure 5D). Interestingly, *ZMYM2* is highly expressed in oxaliplatin acquired-resistant CRC cells and shows a strong correlation with *ANXA9* expression (Figure 5E). Furthermore, CRC patients who exhibit high levels of *ZMYM2* expression also tend to have poorer prognoses, similar to *ANXA9* (Figure 5F). Using ChIP-qPCR, we found that *ZMYM2* binds to the *ANXA9* promoter region (Figure 5G). Notably, this binding is stronger in oxaliplatin acquired-resistant cell lines, suggesting a more pronounced regulatory interaction in the context of chemoresistance (Figure 5H). Functional studies demonstrated that the knockdown of *ZMYM2* by two siRNAs, conformed using WB, significantly suppressed the proliferation and colony formation of CRC cells (Figure 5I,J). Importantly, the knockdown of *ZMYM2* also enhances the sensitivity to oxaliplatin in CRC cells (Figure 5K). These findings thus support the role of *ZMYM2* in promoting tumorigenesis in CRC.

## 4. Discussion

Oxaliplatin-based regimens are a cornerstone in the treatment of CRC; however, the emergence of resistance and subsequent tumor recurrence continue to limit its therapeutic efficacy and patient survival. Elucidating the molecular underpinnings of chemoresistance is thus critical for developing more effective treatment strategies. In this study, we performed a genome-wide transcriptomic analysis to uncover key mediators associated with oxaliplatin resistance in CRC. Among the top candidates, *ANXA9* emerged as a significantly overexpressed gene in chemoresistant tumors, and its expression correlated with a poor prognosis across multiple cancer types. While *ANXA9* has been previously linked to metastasis and immune infiltration in gastrointestinal tumors [16,20,23], its specific role in chemoresistance in CRC had not been clearly defined. Our results provide compelling evidence that *ANXA9* overexpression contributes to oxaliplatin resistance and tumor aggressiveness in CRC.

Consistent with this, an elevated *ANXA9* expression was confirmed in oxaliplatin acquired-resistant CRC cell lines. Both the pharmacological and genetic suppression of *ANXA9* led to a marked decrease in cell proliferation and significantly enhanced oxaliplatin sensitivity in vitro and in vivo. These findings establish *ANXA9* not only as a marker of a poor prognosis but also as a functionally relevant driver of chemoresistance, suggesting that targeting *ANXA9* may provide a novel therapeutic avenue for managing drug-resistant CRC.

To identify therapeutic agents that might overcome *ANXA9*-driven resistance, we conducted a high-throughput drug screen on CRC cells. Among the nine agents with broad inhibitory effects on CRC cell proliferation, G749—a *FLT3* pathway inhibitor—was uniquely effective in downregulating *ANXA9* expression. Though *FLT3* inhibitors have been primarily developed and approved for the treatment of *FLT3*-mutated acute myeloid leukemia (AML) [35], their role in solid tumors, particularly CRC, remains underexplored. Previous studies have demonstrated that *FLT3* is detectable at the mRNA and protein levels in colorectal cancer tissues, despite the rarity of *FLT3* mutations in CRC [36,37]. These findings support the biological plausibility of targeting the *FLT3* pathway in CRC models. Accordingly, the use of G749 in our study is based on the presence of *FLT3* expression rather than mutational activation, providing a rationale for exploring *FLT3* inhibitors as therapeutic agents in CRC. In our study, G749 reduced *ANXA9* expression levels, but its direct effect on upstream regulators such as *ZMYM2* has not been fully established and warrants further investigation.

While G749 effectively downregulated *ANXA9* and overcame oxaliplatin resistance in our models, its specificity to the *FLT3-ZMYM2-ANXA9* axis remains to be fully elucidated. For instance, we did not compare G749 with other *FLT3* inhibitors (e.g., midostaurin or gilteritinib) to confirm whether *ANXA9* suppression is a class effect or unique to G749. Furthermore, kinase inhibitors such as G749 may exhibit off-target effects due to structural similarities across kinases, potentially influencing pathways beyond *FLT3*. Future studies should include kinase profiling and comparative analyses with other *FLT3* inhibitors to validate its specificity.

Notably, *ZMYM2* was found to bind directly to the *ANXA9* promoter, with this interaction being more pronounced in oxaliplatin-resistant CRC cell lines. This provides a mechanistic insight into how *FLT3* pathway modulation may influence *ANXA9* expression and, by extension, therapeutic resistance. The combination of G749 and oxaliplatin demonstrated enhanced anti-tumor efficacy in vitro, in organoid models, and in mouse xenografts, supporting the translational potential of this dual-target strategy.

However, despite these promising findings, several challenges remain. *FLT3* inhibitors, including G749, may exhibit off-target effects and systemic toxicity due to their impact on non-malignant tissues expressing *FLT3* or structurally similar kinases. These safety concerns could limit their clinical application in non-hematological cancers like CRC. Moreover, resistance to *FLT3* inhibitors themselves has been observed in AML through various adaptive mechanisms, raising the possibility of similar resistance pathways in CRC that require careful monitoring.

While our study focused on evaluating the antitumor efficacy of G749, the assessment of systemic toxicity was limited to body weight monitoring. Given the potential off-target effects of kinase inhibitors, future preclinical studies should include comprehensive evaluations of hematologic, hepatic, and renal parameters to fully characterize the safety profile of G749. Therefore, before *FLT3* inhibitors such as G749 can be integrated into CRC treatment protocols, further validation in preclinical models and well-designed randomized controlled trials (RCTs) will be essential. Longitudinal studies should be conducted to evaluate the durability of the therapeutic response, potential toxicities, and biomarkers predictive of a benefit from treatment. The stratification of patients based on *ANXA9* or *ZMYM2* expression may also aid in identifying those most likely to benefit from *FLT3*-targeted strategies.

In summary, our study identifies the *ZMYM2-ANXA9* axis as a key contributor to oxaliplatin resistance in CRC. Although the precise role of *ZMYM2* in mediating G749’s action remains to be clarified, our findings suggest that this axis could serve as a therapeutic vulnerability. Targeting this axis with the *FLT3* inhibitor G749 offers a promising new therapeutic strategy. Nonetheless, the clinical translation of this approach will depend on further mechanistic studies and carefully conducted trials to assess efficacy, safety, and patient selection criteria. The integration of *FLT3* inhibitors into current chemotherapeutic regimens could represent a significant advance in the treatment of chemoresistant CRC.

## Data Availability

The Cancer Genome Atlas (TCGA) pan-cancer data were obtained from UCSC Xena (https://xenabrowser.net/datapages/?cohort=TCGA%20Pan-Cancer%20(PANCAN), (accessed on 12 April 2024)), including gene expression RNA-seq data (https://xenabrowser.net/datapages/?dataset=tcga_RSEM_gene_fpkm&host=https%3A%2F%2Ftoil.xenahubs.net&removeHub=https%3A%2F%2Fxena.treehouse.gi.ucsc.edu%3A443), (accessed on 12 April 2024)), phenotype–sample type and primary disease data (https://xenabrowser.net/datapages/?dataset=TCGA_phenotype_denseDataOnlyDownload.tsv&host=https%3A%2F%2Fpancanatlas.xenahubs.net&removeHub=https%3A%2F%2Fxena.treehouse.gi.ucsc.edu%3A443), (accessed on 12 April 2024)), and phenotype–curated clinical data (https://xenabrowser.net/datapages/?dataset=Survival_SupplementalTable_S1_20171025_xena_sp&host=https%3A%2F%2Fpancanatlas.xenahubs.net&removeHub=https%3A%2F%2Fxena.treehouse.gi.ucsc.edu%3A443) (accessed on 12 April 2024)). Additional RNA-seq data reported in this article have been deposited in NCBI’s Gene Expression Omnibus (GEO) and are accessible through GEO Series accession numbers GSE39582, GSE205035, and GSE207521.

## Supplementary Materials

The following supporting information can be downloaded at: https://www.mdpi.com/article/10.3390/biomedicines13051247/s1, Table S1: Primer and siRNA sequences; Table S2: Correlation of *ANXA9* Protein Expression with Clinicopathological Features in a Cohort of 146 Stage III CRC Patients;Table S3: Cell cycle related compound library; Table S4: Cell cycle related compound library screen in WiDr; Table S5: Cell cycle related compound library screen in DLD1.

## References

[1] Bray F., Ferlay J., Soerjomataram I., Siegel R.L., Torre L.A., Jemal A. (2018). Global cancer statistics 2018: GLOBOCAN estimates of incidence and mortality worldwide for 36 cancers in 185 countries. CA Cancer J. Clin..

[2] Taieb J., Kourie H.R., Emile J.F., Le Malicot K., Balogoun R., Tabernero J., Mini E., Folprecht G., Van Laethem J.L., Mulot C. (2018). Association of Prognostic Value of Primary Tumor Location in Stage III Colon Cancer with RAS and BRAF Mutational Status. JAMA Oncol..

[3] Marin J.J., Sanchez de Medina F., Castaño B., Bujanda L., Romero M.R., Martinez-Augustin O., Moral-Avila R.D., Briz O. (2012). Chemoprevention, chemotherapy, and chemoresistance in colorectal cancer. Drug Metab. Rev..

[4] Tang Y.A., Chen Y.F., Bao Y., Mahara S., Yatim S.M.J.M., Oguz G., Lee P.L., Feng M., Cai Y., Tan E.Y. (2018). Hypoxic tumor microenvironment activates GLI2 via HIF-1α and TGF-β2 to promote chemoresistance in colorectal cancer. Proc. Natl. Acad. Sci. USA.

[5] Tan J., Li Z., Lee P.L., Guan P., Aau M.Y., Lee S.T., Feng M., Lim C.Z., Lee E.Y., Wee Z.N. (2013). PDK1 signaling toward PLK1-MYC activation confers oncogenic transformation, tumor-initiating cell activation, and resistance to mTOR-targeted therapy. Cancer Discov..

[6] Robey R.W., Pluchino K.M., Hall M.D., Fojo A.T., Bates S.E., Gottesman M.M. (2018). Revisiting the role of ABC transporters in multidrug-resistant cancer. Nat. Rev. Cancer.

[7] Fletcher T., Thompson A.J., Ashrafian H., Darzi A. (2022). The measurement and modification of hypoxia in colorectal cancer: Overlooked but not forgotten. Gastroenterol. Rep..

[8] Wood G.E., Hockings H., Hilton D.M., Kermorgant S. (2021). The role of MET in chemotherapy resistance. Oncogene.

[9] Shin J.K., Huh J.W., Lee W.Y., Yun S.H., Kim H.C., Cho Y.B., Park Y.A. (2022). Clinical prediction model of pathological response following neoadjuvant chemoradiotherapy for rectal cancer. Sci. Rep..

[10] Stockton J.D., Tee L., Whalley C., James J., Dilworth M., Wheat R., Nieto T., Geh I., Barros-Silva J.D., S-CORT Consortium (2021). Complete response to neoadjuvant chemoradiotherapy in rectal cancer is associated with RAS/AKT mutations and high tumour mutational burden. Radiat. Oncol..

[11] Cercek A., Dos Santos Fernandes G., Roxburgh C.S., Ganesh K., Ng S., Sanchez-Vega F., Yaeger R., Segal N.H., Reidy-Lagunes D.L., Varghese A.M. (2020). Mismatch Repair-Deficient Rectal Cancer and Resistance to Neoadjuvant Chemotherapy. Clin. Cancer Res..

[12] Yuan Y., Sun W., Xie J., Zhang Z., Luo J., Han X., Xiong Y., Yang Y., Zhang Y. (2025). RNA nanotherapeutics for hepatocellular carcinoma treatment. Theranostics.

[13] Brown J.A., Ni Chonghaile T., Matchett K.B., Lynam-Lennon N., Kiely P.A. (2016). Big Data-Led Cancer Research, Application, and Insights. Cancer Res..

[14] Schloer S., Pajonczyk D., Rescher U. (2018). Annexins in Translational Research: Hidden Treasures to Be Found. Int. J. Mol. Sci..

[15] Mirsaeidi M., Gidfar S., Vu A., Schraufnagel D. (2016). Annexins family: Insights into their functions and potential role in pathogenesis of sarcoidosis. J. Transl. Med..

[16] Yu S., Bian H., Gao X., Gui L. (2018). Annexin A9 promotes invasion and metastasis of colorectal cancer and predicts poor prognosis. Int. J. Mol. Med..

[17] Ma S., Lu C.C., Yang L.Y., Wang J.-J., Wang B.-S., Cai H.-Q., Hao J.-J., Xu X., Cai Y., Zhang Y. (2018). ANXA2 promotes esophageal cancer progression by activating MYC-HIF1A-VEGF axis. J. Exp. Clin. Cancer Res..

[18] Bizzarro V., Belvedere R., Migliaro V., Romano E., Parente L., Petrella A. (2017). Hypoxia regulates ANXA1 expression to support prostate cancer cell invasion and aggressiveness. Cell Adhes. Migr..

[19] Wang K., Li J. (2016). Overexpression of ANXA3 is an independent prognostic indicator in gastric cancer and its depletion suppresses cell proliferation and tumor growth. Oncotarget.

[20] Bello N., Lopez-Kleine L. (2023). Prog-Plot—A visual method to determine functional relationships for false discovery rate regression methods. J. Cell Sci..

[21] Bai F., Zhang P., Fu Y., Chen H., Zhang M., Huang Q., Li D., Li B., Wu K. (2020). Targeting ANXA1 abrogates Treg-mediated immune suppression in triple-negative breast cancer. J. Immunother. Cancer.

[22] Wang Z., Zhou X., Deng X., Ye D., Liu D., Zhou B., Zheng W., Wang X., Wang Y., Borkhuu O. (2023). miR-186-*ANXA9* signaling inhibits tumorigenesis in breast cancer. Front. Oncol..

[23] Zhang T., Yu S., Zhao S. (2021). *ANXA9* as a novel prognostic biomarker associated with immune infiltrates in gastric cancer. PeerJ.

[24] Perl A.E., Martinelli G., Cortes J.E., Neubauer A., Berman E., Paolini S., Montesinos P., Baer M.R., Larson R.A., Ustun C. (2019). Gilteritinib or Chemotherapy for Relapsed or Refractory *FLT3*-Mutated AML. N. Engl. J. Med..

[25] Stone R.M., Mandrekar S.J., Sanford B.L., Laumann K., Geyer S., Bloomfield C.D., Thiede C., Prior T.W., Döhner K., Marcucci G. (2017). Midostaurin plus Chemotherapy for Acute Myeloid Leukemia with a *FLT3* Mutation. N. Engl. J. Med..

[26] Zhang Z., Hu R., Liu J., Yang X., Xiao Y., Xu X., Liu X., Zeng W., Zhang S., Wang L. (2025). Antitumor activity of gilteritinib, an inhibitor of AXL, in human solid tumors. Cell Death Discov..

[27] Renneville A., Gasser J.A., Grinshpun D.E., Jean Beltran P.M., Udeshi N.D., Matyskiela M.E., Clayton T., McConkey M., Viswanathan K., Tepper A. (2021). Avadomide induces degradation of *ZMYM2* fusion oncoproteins in hematologic malignancies. Blood Cancer Discov..

[28] Aydin E., Tokat U.M., Adibi A., Ozgu E., Bilgic S.N., Demiray M. (2024). Case report: Precision guided reactive cancer management: Molecular complete response in heavily pretreated metastatic CRC by dual immunotherapy and sorafenib. Front. Oncol..

[29] Munthe-Kaas M.C., Forthun R.B., Brendehaug A., Eek A.K.M., Høysæter T.B., Osnes L.T., Prescott T., Spetalen S., Hovland R. (2021). Partial Response to Sorafenib in a Child with a Myeloid/Lymphoid Neoplasm, Eosinophilia, and a *ZMYM2-FLT3* Fusion. J. Pediatr. Hematol. Oncol..

[30] Graham-Paquin A.L., Saini D., Sirois J., Hossain I., Katz M.S., Zhuang Q.K.-W., Kwon S.Y., Yamanaka Y., Bourque G., Bouchard M. (2023). *ZMYM2* is essential for methylation of germline genes and active transposons in embryonic development. Nucleic Acids Res..

[31] Edge S.B., Compton C.C. (2010). The American Joint Committee on Cancer: The 7th edition of the AJCC cancer staging manual and the future of TNM. Ann. Surg. Oncol..

[32] Yu Z., Deng P., Chen Y., Lin D., Liu S., Hong J., Guan P., Chen J., Zhong M.-E., Chen J. (2024). Pharmacological modulation of RB1 activity mitigates resistance to neoadjuvant chemotherapy in locally advanced rectal cancer. Proc. Natl. Acad. Sci. USA.

[33] Liu N., Cui R.X., He Q.M., Huang B.J., Sun Y., Xie D., Zeng J., Wang H.Y., Ma J. (2013). Reduced expression of Dicer11 is associated with poor prognosis in patients with nasopharyngeal carcinoma. Med. Oncol..

[34] Jie X., Fong W.P., Zhou R., Zhao Y., Zhao Y., Meng R., Zhang S., Dong X., Zhang T., Yang K. (2021). USP9X-mediated KDM4C deubiquitination promotes lung cancer radioresistance by epigenetically inducing TGF-β2 transcription. Cell Death Differ..

[35] Daver N., Schlenk R.F., Russell N.H., Levis M.J. (2019). Targeting *FLT3* mutations in AML: Review of current knowledge and evidence. Leukemia.

[36] Hasegawa H., Taniguchi H., Nakamura Y., Kato T., Fujii S., Ebi H., Shiozawa M., Yuki S., Masuishi T., Kato K. (2021). FMS-like tyrosine kinase 3 (*FLT3*) amplification in patients with metastatic colorectal cancer. Cancer Sci..

[37] Sanchez-Vega F., Mina M., Armenia J., Chatila W.K., Luna A., La K.C., Dimitriadoy S., Liu D.L., Kantheti H.S., Saghafinia S. (2018). Oncogenic Signaling Pathways in The Cancer Genome Atlas. Cell.

