# Supplementary material for: Targeting the ZMYM2-ANXA9 Axis with FLT3 Inhibitor G749 Overcomes Oxaliplatin Resistance in Colorectal Cancer
Source: Biomedicines. 2025 May 20;13(5):1247. doi: 10.3390/biomedicines13051247 (PMC12108716; doi:10.3390/biomedicines13051247)
Supplement: Supplementary file 1 [file biomedicines-13-01247-s001.zip › Table S1.pdf]

Primer and siRNA sequences

Category	Target	Direction	Sequence (5' → 3')
ChIP-qPCR	ANXA9 #1	Forward	TGCCTGGCCACATTACCTTA
		Reverse	GCTTCGCTAGTCCTTTCAGC
	ANXA9 #2	Forward	ACTAGGTCTCTGTGGTCCCA
		Reverse	GGCTGTACAGAGTCCCACAT
siRNA (RNAi)	ANXA9	siRNA #1	CCTGGCAGTCTACAAACACAA
		siRNA #2	CCCAATTACCAAGTCCTGATT
	ZMYM2	siRNA #1	GCTGGGTATTACCATGTAAAT
		siRNA #2	GCGAAACTCTTTACCTCAATA
RT-qPCR	ANXA9	Forward	CAGCTCATCTCACGAAACTTCC
		Reverse	GGTTCGAGTGGCAAGAATTTCAA
	ZMYM2	Forward	TCCCCGTAATCAGAAACAACCA
		Reverse	TGGACAGACGGCTGGAAAATC
	β-actin	Forward	CACCATTGGCAATGAGCGGTTC
		Reverse	AGGTCTTTGCGGATGTCCACGT
