# Supplementary material for: Targeting the ZMYM2-ANXA9 Axis with FLT3 Inhibitor G749 Overcomes Oxaliplatin Resistance in Colorectal Cancer
Source: Biomedicines. 2025 May 20;13(5):1247. doi: 10.3390/biomedicines13051247 (PMC12108716; doi:10.3390/biomedicines13051247)
Supplement: Supplementary file 1 [file biomedicines-13-01247-s001.zip › Table S2.pdf]

Table S2 Correlation of ANXA9 Protein Expression with Clinicopathological Features in a Cohort of 146 Stage III CRC Patients

Clinical characteristics	N=146	Low expression (n=73)	High expression (n=73)	P
Sex				0.740
Male	68/146 (46.6%)	33/73 (45.2%)	35/73 (47.9%)	
Female	78/146 (53.4%)	40/73 (54.8%)	38/73 (52.1%)	
Age (years)				0.680
<60	91/146 (62.3%)	44/73 (60.3%)	47/73 (64.4%)	
60≥	55/146 (37.7%)	29/73 (39.7%)	26/73 (35.6%)	
BMI (kg/m^2)				1.000
<25	112/146 (76.7%)	56/73 (76.7%)	56/73 (76.7%)	
25≥	34/146 (23.3%)	17/73 (23.3%)	17/73 (23.3%)	
T				0.716
T3+T4	138/146(94.5%)	70/73(95.9%)	68/73(93.2%)	
T1+T2	8/146(5.5%)	3/73(4.1%)	5 /73(6.8%)	
N				0.350
1	107/146(73.3%)	56/73(76.7%)	51/73(69.9%)	
2	39/146(26.7%)	17/73(23.3%)	22/73(30.1%)	
Tissue Typing				1.000
Adenocarcinoma	136/146(93.2%)	68/73(93.2%)	68/73(93.2%)	
Non-adenocarcinoma	10/146(6.8%)	5/73(6.8%)	5/73(6.8%)	
Tumor differentiation				0.578
Poorly differentiated (undifferentiated)	10/146(6.8%)	5/73(6.8%)	5/73(6.8%)	
Moderately differentiated	107/146(73.3%)	56/73(76.7%)	51/73(69.9%)	
Well differentiated	29/146(19.9%)	12/73(16.4%)	17/73(23.3%)	
CEA(ng/ml)				0.240
≤5.0	85/146(58.2%)	39/73(53.4%)	46/73(63.0%)	
>5.0	61/146(41.8%)	34/73(46.6%)	27/73(37.0%)	
CA-199(U/ml)				0.062
≤37.0	117/146(80.1%)	54/73(74.0%)	63/73(86.3%)	
>37.0	29/146(19.9%)	19/73(26.0%)	10/73(13.7%)	
CA125(U/ml)				0.384
≤35.0	133/146(91.1%)	65/73(89.0%)	68/73(93.2%)	
>35.0	13/146(8.9%)	8/73(11.0%)	5/73(6.5%)	
Postoperative radiotherapy				0.752
No	144/146 (98.6%)	72/73(98.6%)	72/73(98.6%)	
Yes	2/146(1.4%)	1/73(1.4%)	1/73(1.4%)	
Postoperative chemotherapy				/
No	0	0	0	
Yes	146/146(100.0%)	73/73 (100.0%)	73/73 (100.1%)	
dMMR				0.615
No	129/146(88.4%)	66/73(90.4%)	63/73(86.3%)	
Yes	17/146(11.6%)	7/73(9.6%)	10/73(13.7%)	
Metastasis or recurrence				***0.007***
No	122/146(83.6%)	67/73 (91.8%)	55/73 (75.3%)	
Yes	24/146(16.4%)	6/73 (8.2%)	18/73 (24.7%)	
Death				0.363
No	141/146(96.6%)	72/73 (98.6%)	69/73 (94.5%)	
Yes	5/146(3.4%)	1/73 (1.4%)	4/73 (5.5%)	
